# Severe Painful Vaso-Occlusive Crises and Mortality in a Contemporary Adult Sickle Cell Anemia Cohort Study

**DOI:** 10.1371/journal.pone.0079923

**Published:** 2013-11-05

**Authors:** Deepika S. Darbari, Zhengyuan Wang, Minjung Kwak, Mariana Hildesheim, James Nichols, Darlene Allen, Catherine Seamon, Marlene Peters-Lawrence, Anna Conrey, Mary K. Hall, Gregory J. Kato, James G. Taylor VI

**Affiliations:** 1 Center for Cancer and Blood Disorders, Children’s National Medical Center, Washington DC, United States of America; 2 Genomic Medicine Section, Hematology Branch, National Heart, Lung and Blood Institute, National Institutes of Health, Bethesda, Maryland, United States of America; 3 Office of Biostatistics Research, National Heart, Lung and Blood Institute, National Institutes of Health, Bethesda, Maryland, United States of America; 4 Sickle Cell Disease Vascular Section, Hematology Branch, National Heart, Lung and Blood Institute, National Institutes of Health, Bethesda, Maryland, United States of America; 5 Critical Care Medicine, Clinical Center, National Institutes of Health, Bethesda, Maryland, United States of America; Glaxo Smith Kline, Denmark

## Abstract

**Background:**

Frequent painful vaso-occlusive crises (VOCs) were associated with mortality in the Cooperative Study of Sickle Cell Disease (CSSCD) over twenty years ago. Modern therapies for sickle cell anemia (SCA) like hydroxyurea are believed to have improved overall patient survival. The current study sought to determine the relevance of the association between more frequent VOCs and death and its relative impact upon overall mortality compared to other known risk factors in a contemporary adult SCA cohort.

**Methods:**

Two hundred sixty four SCA adults were assigned into two groups based on patient reported outcomes for emergency department (ED) visits or hospitalizations for painful VOC treatment during the 12 months prior to evaluation.

**Results:**

Higher baseline hematocrit (p = 0.0008), ferritin (p = 0.005), and HDL cholesterol (p = 0.01) were independently associated with 1 or more painful VOCs requiring an ED visit or hospitalization for acute pain. During a median follow-up of 5 years, mortality was higher in the ED visit/hospitalization group (relative risk [RR] 2.68, 95% CI 1.1-6.5, p = 0.03). Higher tricuspid regurgitatant jet velocity (TRV) (RR 2.41, 95% CI 1.5-3.9, p < 0.0001), elevated ferritin (RR 4.00, 95% CI 1.8-9.0, p = 0.001) and lower glomerular filtration rate (RR=2.73, 95% CI 1.6-4.6, p < 0.0001) were also independent risk factors for mortality.

**Conclusions:**

Severe painful VOCs remain a marker for SCA disease severity and premature mortality in a modern cohort along with other known risk factors for death including high TRV, high ferritin and lower renal function. The number of patient reported pain crises requiring healthcare utilization is an easily obtained outcome that could help to identify high risk patients for disease modifying therapies.

**Trial Registration:**

ClinicalTrials.gov NCT00011648 http://clinicaltrials.gov/

## Introduction

Sickle cell disease (SCD) is the most common monogenic disease in the United States, resulting from mutations in the beta-subunit of the hemoglobin molecule. Acute episodes of pain or vaso-occlusive crises (VOCs) are a protean hallmark of SCD with economic impact due to the cost of unscheduled health care[1]. Acute VOCs are due to erythrocyte microvascular occlusion and tissue hypoxia, especially in sickle cell anemia (SCA)[2]. Inter-individual differences in severe VOCs leading to utilization of acute care are more common in a sub-group with frequent pain[3–6]. Reasons for this variability have not been elucidated, but higher hematocrit and lower fetal hemoglobin (HbF) are strong predictors for frequent VOCs[4]. Frequent VOCs defined by physician treatment and a duration of more than 2 hours were associated with higher mortality in the Cooperative Study of Sickle Cell Disease (CSSCD) although this association has not been evaluated in contemporary populations where health care utilization for acute painful episodes remains common despite the availability of SCA specific treatments[3,4,7]. 

 SCA is characterized by specific complications that are risk factors for early death, although modern therapies are likely to have altered the natural history and mortality of SCA[8–10]. In particular, hydroxyurea (HU) in SCA is an effective medication to significantly reduce hospitalizations for pain[9,11]. Scheduled chronic transfusion in children at high risk for stroke prevents both cerebrovascular events and reduces the prevalence of VOCs[8,12]. Additionally, an elevated tricuspid regurgitant velocity (TRV) is a risk factor for death in some series and suggestive of cardiopulmonary disease, although trials of pulmonary vasodilators have failed to demonstrate a clinical benefit[13–17]. Based upon these observations, we hypothesized that therapies with demonstrated SCA efficacy like transfusion programs and HU have altered the magnitude of associations between acute painful episodes, health care utilization and mortality. We sought to 1) identify markers associated with VOCs for comparison to established VOC associations with baseline hemoglobin and HbF expression and 2) determine if acute painful episodes defined by hospital based treatment as a patient reported outcome (PRO) are a relevant measure of disease severity and risk for death in a contemporary SCA cohort at a single referral institution.

## Methods

### Study population

Subjects 18 years of age or older were enrolled according to a protocol for evaluation of adults with SCD after written informed consent (The Bethesda Sickle Cell Cohort Study, ClinicalTrials.gov identifier NCT00011648) between 2001-2007[14,18]. The study was conducted in accordance with the Declaration of Helsinki and was approved by the National Heart, Lung and Blood Institute Institutional Review Board. Recruitment was through advertisement and community outreach to local clinics, providers, advocacy groups and patients. Those receiving modern therapies like transfusions and HU were not excluded. Subjects with any acute complication in the 2 weeks prior to evaluation were not enrolled, although recruitment was permitted at a later date. Data collection included a history, physical examination and transthoracic echocardiogram as previously described[14,18]. Steady state hemoglobin, fetal hemoglobin (HbF), leukocytes, platelets, reticulocytes, serum lactate dehydrogenase (LDH), aspartate aminotransferase, bilirubin and ferritin were determined at the time of enrollment using standard methodologies. Subjects were surveyed for the number of severe acute painful events requiring treatment by a physician in an emergency department or as a hospital inpatient during the 12 months prior to evaluation as a PRO. After enrollment, subjects were contacted at 2 year intervals through 2010 to document survival. Death certificates and the Social Security Death Master File were used for additional documentation.

### Observations, patient reported outcomes and definitions

Analysis of VOCs was restricted to subjects with SCA where the diagnosis was made by DNA sequencing, hemoglobin electrophoresis and/or high-performance liquid chromatography[18]. The presence of co-existing α-thalassemia was determined by multiplex polymerase chain reactions as previously described[18,19]. “Severe” VOCs were defined as patient reported emergency department visits or hospitalizations for pain treatment during the 12 months prior to enrollment[20]. Neither painful episodes occurring at home without medical intervention nor hospitalizations for other SCA complications without acute pain were included in this definition. HU therapy was recorded dichotomously based upon treatment decisions made by primary healthcare providers. Glomerular filtration rate (GFR) was calculated using the abbreviated Modification of Diet in Renal Disease (MDRD) equation[21].

### Pain history and group assignment

For this report, the number of PRO painful VOCs, as defined above, were used to categorize subjects into 2 groups: low VOC (0 emergency department visits/hospitalizations for pain) and high VOC (≥1 events during the prior 12 months) groups[4]. The high pain group was compared to all others. 

### Statistical analysis

Continuous data comparisons were by Wilcoxon or Pearson’s χ^2^ test between VOC groups with p values. Using selected variables significant by univariate analysis, we identified a logistic regression model by stepwise backward elimination where all variables had an α ≤ 0.05. Regression odds ratios compared the 75^th^ relative to 25^th^ percentiles for continuous variables calculated as e^(coefficient)(75th percentile – 25th percentile)^. Survival analyses utilized Kaplan-Meier and Cox proportional hazards modeling. The proportional hazards assumption was met as tested by the linearity of the rescales Schoenfeld residuals of the time dependent covariates. Two sided α’s ≤ 0.05 defined statistical significance with Bonferroni correction are presented for comparison. Analyses were performed with Prism (Graph Pad Software, San Diego, CA), R (http://CRAN.R-project.org), SAS 9.1.3 (Cary, NC), STATA 9.0 (College Station, TX) and SVS (Golden Helix, Bozeman, MT). 

## Results

### Participants

Two hundred sixty four SCA subjects with VOC PRO data were available for analysis in the current study from a population of 400 SCD subjects evaluated. The median age of SCA participants was 32 years and 126 (47%) were male. Forty one percent of individuals reported HU treatment and 39 % reported receiving more than 10 red cell transfusions during their lifetime.

### Severe painful episodes and clinical / laboratory characteristics

The characteristics of the SCA VOC groups are shown in Table 1. One hundred and sixty study participants (60%) reported at least one severe VOC requiring hospital based treatment in the 12 months preceding enrollment and 22% of individuals in this group reported 5 or more such events. A patient reported history of asthma, acute chest syndrome, and priapism was not different between VOC groups, nor was the proportion of individuals with concurrent α-thalassemia. Although not statistically significant, a higher proportion of study participants in the high pain crisis group were on HU, more likely to have received > 10 red cell transfusions and have had 1 or more episodes of acute chest syndrome. Individuals in the frequent ED/hospitalization group had higher median hemoglobin concentration (9.2 vs. 8.4 g/dL; P=0.0007) and serum ferritin (618 vs. 313 mg/L; P=0.009), while serum lactate dehydrogenase (LDH) (346 vs. 378 U/L; P=0.004) and uric acid (5.5 vs. 6.20 mg/dL; P=0.03) were lower. Fetal hemoglobin concentration was not different between the groups (7.7 vs. 6.9%; P=0.3). The proportion of study subjects with an elevated tricuspid regurgitation velocity (TRV) ≥ 2.5 m/sec, a previously described marker of suspected cardiopulmonary disease in adults with SCA[8,13], was not different between the groups. We further compared the 2 pain crisis sub-groups with 1-4 ED visits/hopitalizations and greater than 4 ED visits/hopitalizations to those with less frequent events (Table S1). Comparison of these 3 groups showed significant differences for hemoglobin concentration (P<0.0001), ferritin (P=0.02) and LDH (P=0.009), although none were not significantly different by pairwise comparison of the 2 pain crisis groups suggesting that further sub-classification of the pain crisis groups is not helpful for identifying additional associations with VOCs. As shown in Table 2, higher hemoglobin (OR 2.04, 95% CI: 1.3-3.1, P=0.001), serum ferritin (OR 2.2, 95% CI: 1.3-3.7, P=0.003) and HDL cholesterol (OR 1.56, 95% CI: 1.1-2.2, P=0.01) were independently associated with 1 or more severe painful episodes that required an ED visit or hospitalization by multiple logistic regression analysis.

**Table 1:** Characteristics of an adult SCA cohort by patient reported severe VOC requiring ED visit/ hospitalization in the past year.

**Parameter**	**Subcategory**	**No ED / Hospitalizations**		**≥1 ED / Hospitalizations**		P Value*
		**N with data**	**N or Median**	**N with data**	**N or Median**	
Age, years (IQR)		104	33.5 (25.5-46.0)	160	31.0 (25.0-40.0)	0.1
Sex, N (%)		104		160		0.7
	Male		51 (49.0)		75 (46.9)	
	Female		53 (51.0)		85 (53.1)	
Hydroxyurea, N (%)		100	38 (38.0)	158	69 (43.7)	0.4
Lifetime transfusions, N (%)		99		136		0.1
	0		10 (10.1)		6 (4.4)	
	1-10		52 (52.5)		64 (47.1)	
	>10		37 (37.4)		66 (48.5)	
α- thalassemia (carrier or trait), N (%)		77	22 (28.6)	130	37 (28.5)	0.9
Asthma, N (%)		101	16 (15.8)	158	26 (16.5)	0.9
Acute chest syndrome, N (%)		102	79 (77.5)	157	135 (86.0)	0.08
Priapism, N (%)		48	22 (45.8)	75	33 (44.0)	0.8
TRV ≥ 2.5, m/sec., N (%)‡		95	56 (59.0)	138	65 (47.1)	0.08
TRV ≥ 3.0, m/sec., N (%)‡		95	22 (23.2)	138	21 (15.2)	0.1
WBC, 10^9^/L (IQR)		100	10.4 (8.5-12.1)	155	10.4 (8.7-12.6)	0.5
Hematocrit, % (IQR)		100	24.6 (21.0-28.1)	155	26.9 (24.3-29.3)	0.0003†
Fetal hemoglobin, % (IQR)		94	6.9 (2.9-11.5)	151	7.7 (3.4-13.5)	0.3
Ferritin, μg/L (IQR)		96	313 (107-1,052)	145	618 (199-1,569)	0.009
Lactate dehydrogenase, U/L (IQR)		94	378 (302-495)	135	346 (256-416)	0.004
Aspartate aminotransferase, U/L (IQR)		99	42 (33.0-51.0)	152	38 (28-54)	0.2
Total bilirubin, mg/dL (IQR)		99	3.1 (1.9-4.1)	152	2.6 (1.7-3.8)	0.2
Uric acid, mg/dL (IQR)		99	6.2 (5.3-7.9)	154	5.9 (4.6-7.3)	0.03
High density lipoprotein cholesterol, mg/dL (IQR)		89	36 (31-43)	142	39 (33-47)	0.02
C-reactive protein, mg/dL (IQR)		88	0.20 (0.20-0.74)	140	0.31 (0.20-0.79)	0.7
Glomerular filtration rate, mL/min./1.73m^2^ (IQR)		101	160.6 (103.3-199.6)	155	162.3 (122.3-203.4)	0.4

From Wilcoxon or Pearson’s χ^2^ test between groups of ED visits/Hospitalizations.

^†^ P= < 0.05 after Bonforoni correction

^‡^ Subjects with an undetectable TRV by echocardiogram were excluded from this analysis (N=5 in the No ED/Hospitalization Group and N=16 in the >1 ED/Hospitalization Group).

Abbreviations: ED, emergency department; IQR, interquartile range; SCA, sickle cell anemia; TRV, tricuspid regurgitant jet velocity; VOC, vaso-occlusive crisis; WBC, white blood cell count.

**Table 2:** Characteristics associated with patient reported severe VOCs by multivariable logistic regression.

**Independent variable** *	**Coefficient**	**OR** (**95% CI**) †	**p Value**
Hematocrit	0.51	1.99 (1.46-2.71)	0.0008
Ferritin	0.45	1.83 (1.34-2.49)	0.005
HDL-Cholesterol	0.35	1.61 (1.20-2.16)	0.01

* Covariates identified in Table 1 or prior studies (7,9) were included in multivariable regression models with stepwise elimination of variables that were not significant in the final model.

^†^ Odds ratio is given for the 75^th^ relative to 25^th^ percentile for continuous variables calculated as e^(coefficient)(75th percentile – 25th percentile)^.

Abbreviations: OR, odds ratio; HDL, high density lipoprotein; VOC, vaso-occlusive crisis.

### Patient reported severe pain crises are an independent risk factor for mortality in adults with SCA

**Figure 1:**
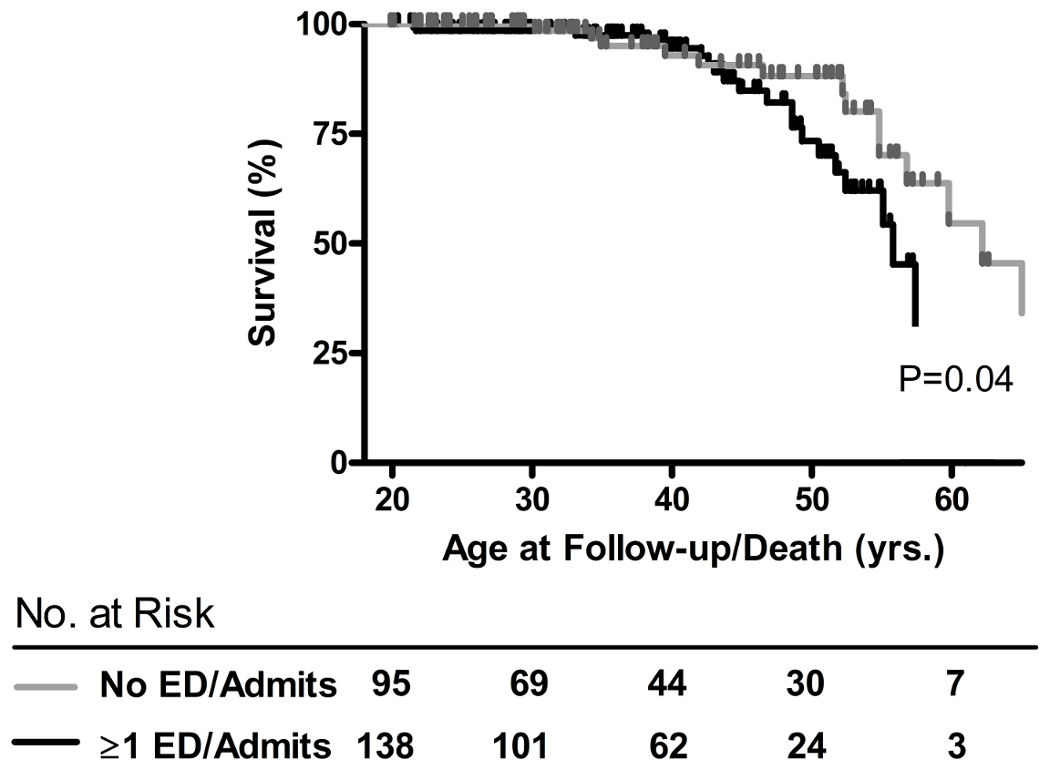
Kaplan Meier (KM) curve showing survival in sickle cell anemia by severe pain crises requiring an ED visit/ hospitalization in past year.

To determine if severe pain crises remain a risk factor for early mortality in contemporary SCA patients, univariate survival analysis was performed. During a median follow-up of 4.92 years, 40 (15.1%) subjects died and 9 (3.4%) were lost to follow-up. Kaplan-Meier showed a younger age at death in the high VOC group (55.8 vs. 66.2 years; P=0.04) (Figure 1). Kaplan-Meier also showed a significant difference when comparing 3 pain sub-groups (0 ED visits/hospitalizations, 1-4 ED visits/hopitalizations and ≥5 ED visits/hospitalizations) (P=0.02 for trend, Figure S1). Because the Kaplan Meier curves comparing the 2 pain groups (1-4 versus ≥5 ED visits/hospitalizations) were not significantly different (P=0.22, Figure S1), a Cox proportional hazards analysis of mortality utilized the categorical high (≥1 ED visits/hospitalizations) versus low VOC group classification. The risk of death among 208 evaluable subjects with 35 deaths during follow-up by proportional hazards modeling was again significantly higher with more than 1 severe VOC during the past 12 months (relative risk [RR] 2.68, P= 0.03), a higher TRV (RR 2.41, P<0.0001), elevated ferritin (RR 4.00, P=0.001) and low estimated glomerular filtration rate (RR 2.73, P<0.0001) (Table 3).

**Table 3:** Cox proportional hazards mortality in adults with SCA.

**Risk factor**	**RR** (**95% CI**)*	**p Value** †
ED/Hospitalization, ≥1 in past year	2.68 (1.1-6.5)	0.03
TRV‡	2.41 (1.5-3.9)	<0.0001
Ferritin	4.00 (1.8-9.0)	0.001
GFR	2.73 (1.6-4.6)	<0.0001

* RR for ED/hospitalization is given relative to no visits in the past year. RR for TRV, ferritin and GFR are given for the 75^th^ relative to the 25^th^ percentile.

^†^ From Wald χ^2^.

^‡^ 21 subjects with undetectable TRV by echocardiogram were excluded from analysis. TRV boundaries for the 75^th^ and 25^th^ percentiles were 2.8 m/s and 2.2 m/s, respectively.

Abbreviations: CI, confidence interval; ED, emergency department; GFR, Modification of Diet in Renal Disease estimated glomerular filtration rate; RR, relative risk; SCA, sickle cell anemia; TRV, tricuspid regurgitatant jet velocity.

## Discussion

We examined VOCs with healthcare utilization and their association with mortality in a contemporary SCA cohort with a high prevalence of HU treatment, a therapy that was not available to subjects included in the first report of this association[4]. We also describe clinical and laboratory characteristics associated with patient reported severe VOCs resulting in an ED visit or hospitalization. Although other SCD genotypes (hemoglobin SC and S beta plus thalassemia) experience vaso-occlusive pain, the present analysis was restricted to sickle cell anemia (homozygous hemoglobin S) due to well known differences in laboratory parameters and the frequency of pain between these genotypes[4,7,22]. In our cohort, about 40% of individuals with SCA reported the absence of hospital admissions or acute treatment in an ED for severe VOCs in the 12 months prior to their initial evaluation on this study, while 22 % reported 5 or more ED visits or hospitalizations during the same time period. These findings highlight the variability of severe painful VOCs within a single genotype despite modern availability of SCD specific therapies. 

Despite reports of overall improved survival in SCD, VOC remains associated with premature death similar to results of the CSSCD and likely remains a suitable marker of disease severity[4,7]. The association of higher steady state hemoglobin with VOCs again replicates a key observation from the CSSCD, likely due to the effect of higher viscosity in promoting microvascular sludging and vaso-occlusion[23,24]. The association of higher LDH with the no ED visit/hospitalization group shows a strong negative correlation between LDH and hemoglobin, although their effects were not independent of one another by logistic regression. Our finding of higher iron burden in the ED visit/ hospitalization group could reflect the higher number of red cell transfusions reported by this group. Worsening of anemia is a frequently observed complication of acute painful VOCs which is often treated with red cell transfusions in a hospital setting. High ferritin could also be a reflection of a high inflammatory state in this group, although other inflammatory markers were not significantly elevated in this high VOC group. Finally, higher HDL cholesterol was independently associated with more frequent VOCs. Hypocholesterolemia is common in SCD and other anemias and is thought to be a manifestation of increased erythroid proliferation[25]. Formation of erythroid cell membranes requires cholesterol, and thus, elevated serum HDL cholesterol may be a marker of less marrow activity in those with SCA and a higher hematocrit[26,27]. Additional studies will be required to determine the mechanism of hypocholestorolemia in SCA and if this marker is relevant to the pathophysiology of vaso-occlusion.

Contrary to the anticipated association, fetal hemoglobin concentration was not significantly higher in the no ED visit / hospitalization group. The antimetabolite HU is effective for reducing complications of SCA including frequent severe painful VOCs, with one of the proposed mechanisms of action being increased fetal hemoglobin production, which in turn reduces sickle hemoglobin polymerization and vaso-occlusion[9]. The absence of this fetal hemoglobin association could be due to many factors including confounding by HU indication, which is an intrinsic limitation of observational studies. Other potential confounding variables not assessed in this study include adequacy of HU dosing, duration of HU therapy and compliance with its use. However, the absence of an association between higher fetal hemoglobin and fewer VOCs has also been reported in a contemporary pediatric SCA cohort that was not treated with HU[28].

The association between painful VOC ED visits and hospitalizations and mortality as reported by the CSSCD almost 20 years ago still remains significant in the contemporary era of SCD specific therapies. Additionally, our analysis shows that more frequent severe VOCs requiring healthcare utilization are associated with mortality independent of other known risk factors including an elevated TRV, renal insufficiency and suspected iron burden. These factors could reflect independent aspects of severe disease and chronic organ damage in the VOC group. We are unable to comment on the causes of death in these individuals since most of the participants were followed and received care in their local emergency departments or hospitals. Our results suggest that individuals with a history of health care utilization for severe pain crises and the presence of any of above mortality risk factors are at a particularly high risk for death and should be treated aggressively with appropriate therapies. 

One limitation of this study was the examination of patient reported outcomes (severe VOCs), which could introduce the bias of self-recall and under or overestimation of the number of actual ED visits/ hospital admissions. Daily pain experienced by ambulatory adults is underestimated and often managed at home; therefore ambulatory pain events are not captured by utilization definitions[6]. The presence of such pattern in our study could lead to underestimation of frequency of severe VOC pain crises in our population. Despite these limitations associated with severe pain crisis as a patient reported outcome, this definition of pain in SCA (i.e. the pain crisis rate) has also been useful as a clinical endpoint in drug trials, as a patient reported component of a forthcoming quality of life measure for adults with SCD (ASCQ-Me or Adult Sickle Cell Quality of Life Measurement Information System) and as a prognostic marker for mortality in the CSSCD[7,9,16,29,30]. The pain crisis rate may also represent a heritable phenotype for studies of genetic modifiers in SCA[31]. To some extent, this validates our use of cost effective, patient reported outcomes for a history of pain to assess SCA disease severity. 

Our findings suggest that the above lab studies are biomarkers indicative of SCA clinical severity that contributes to premature mortality in contemporary patients receiving modern therapies. Although these associations might represent epiphenomena of clinical severity, they provide several hypothetical targets for future investigation of interventions to improve longevity in SCA.

## Supporting Information

Table S1 **Characteristics of an adult SCA cohort by patient reported severe VOC requiring ED visit/ hospitalization in the past year.**
(DOCX)

Figure S1 **Kaplan Meier (KM) curve showing survival in sickle cell anemia is associated with the overall number of pain crisis events defined by ED visits and hospitalizations, but not sub-groups with more frequent occurrences.**
(DOCX)
