# Supplementary material for: Severe Painful Vaso-Occlusive Crises and Mortality in a Contemporary Adult Sickle Cell Anemia Cohort Study
Source: PLoS One. 2013 Nov 5;8(11):e79923. doi: 10.1371/journal.pone.0079923 (PMC3818240; doi:10.1371/journal.pone.0079923)
Supplement: Table S1 — Characteristics of an adult SCA cohort by patient reported severe VOC requiring ED visit/ hospitalization in the past year. (DOCX) [file pone.0079923.s001.docx]

| **Parameter** | **No ED / Hospitalizations** | | **1-4 ED / Hospitalizations** | | **≥5 ED / Hospitalizations** | | **P Value*** |
| --- | --- | --- | --- | --- | --- | --- | --- |
|  | **N with data** | **N or Median** | **N with data** | **N or Median** | **N with data** | **N or Median** |  |
| Age, years (IQR) | 104 | 33.5 (25.5-46.0) | 101 | 33.0 (26.0-42.0) | 59 | 29.0 (23.0-33.5) | 0.053 |
| Male, N (%) | 104 | 51 (49.0) | 101 | 45 (44.6) | 59 | 29 (49.1) | 0.7 |
| Hydroxyurea, N (%) | 100 | 38 (38.0) | 100 | 44 (44.0) | 58 | 25 (43.1) | 0.7 |
| Lifetime transfusions, N (%)  0 / 1-10 / >10 | 99 | 10 (10.1)  52 (52.5)  37 (37.4) | 84 | 5 (6.0)  44 (52.4)  35 (41.7) | 52 | 1 (1.9)  20 (38.5)  31 (59.6) | 0.053 |
| α- thalassemia (carrier or trait), N (%) | 77 | 22 (28.6) | 75 | 24 (32.0) | 50 | 16 (32.0) | 0.9 |
| Asthma, N (%) | 101 | 16 (15.8) | 99 | 12 (12.1) | 59 | 14 (23.7) | 0.2 |
| Acute chest syndrome, N (%) | 102 | 79 (77.5) | 98 | 82 (83.7) | 59 | 53 (89.8) | 0.1 |
| Priapism, N (%) | 48 | 22 (45.8) | 45 | 19 (42.2) | 30 | 14 (46.7) | 0.9 |
| TRV ≥ 2.5, m/sec., N (%)‡ | 95 | 56 (59.0) | 89 | 39 (43.8) | 49 | 26 (53.1) | 0.1 |
| TRV ≥ 3.0, m/sec., N (%)‡ | 95 | 22 (23.2) | 89 | 12 (13.5) | 49 | 9 (18.4) | 0.2 |
| WBC, 10^9^/L (IQR) | 100 | 10.4 (8.5-12.1) | 99 | 9.9 (8.6-12.1) | 56 | 10.9 (8.8-12.7) | 0.3 |
| Hematocrit, % (IQR) | 100 | 24.4 (21.0-28.1) | 59 | 29.0 (23.0-35.5) | 56 | 27.5 (24.5-29.9) | <0.0001† |
| Fetal hemoglobin, % (IQR) | 94 | 6.9 (2.9-11.5) | 94 | 7.5 (3.5-13.4) | 56 | 8.2 (3.6-12.4) | 0.5 |
| Ferritin, μg/L (IQR) | 96 | 313 (107-1,052) | 93 | 524 (210-1,345) | 52 | 653 (184-2611) | 0.02 |
| Lactate dehydrogenase, U/L (IQR) | 94 | 378 (302-495) | 84 | 364 (254-438) | 51 | 331 (283-378) | 0.009 |
| Aspartate aminotransferase, U/L (IQR) | 99 | 42 (33.0-51.0) | 95 | 37 (27-52) | 57 | 40 (30-55) | 0.4 |
| Total bilirubin, mg/dL (IQR) | 99 | 3.1 (1.9-4.1) | 95 | 2.6 (1.9-3.7) | 57 | 2.6 (1.6-3.8) | 0.4 |
| Uric acid, mg/dL (IQR) | 99 | 6.2 (5.3-7.9) | 96 | 5.9 (4.8-7.1) | 58 | 6.0 (4.5-7.9) | 0.09 |
| High density lipoprotein cholesterol, mg/dL (IQR) | 89 | 36 (31-43) | 91 | 39 (34-45) | 51 | 39 (33-51) | 0.052 |
| C-reactive protein, mg/dL (IQR) | 88 | 0.20 (0.20-0.74) | 88 | 0.42 (0.20-0.80) | 52 | 0.20 (0.20-0.78) | 0.9 |
| Glomerular filtration rate, mL/min./1.73m^2^ (IQR) | 101 | 160.6 (103.3-199.6) | 96 | 160.0 (110.3-195.0) | 59 | 170.6 (135.1-219.8) | 0.2 |

* From Kruskal-Wallis test (Nonparametric ANOVA; continuous variables) or Chi square test (categorical variables) between 3 ED visit/Hospitalization groups.

† P= < 0.05 after Bonforoni correction. Post-test P values: 0 vs. 1-4 P<0.001; 0 vs. >5 P<0.01; 1-4 vs. >5 P=NS.

‡ Subjects with an undetectable TRV by echocardiogram were excluded from this analysis (N=21).

Pairwise subgroup comparisons were not significant for age and HDL cholesterol.

Pairwise comparison for ferritin, LDH and transfusion history from <1 to >5 were P<0.05; other pairwise comparisons were not significant.

Abbreviations: ED, emergency department; IQR, interquartile range; SCA, sickle cell anemia; TRV, tricuspid regurgitant jet velocity; VOC, vaso-occlusive crisis; WBC, white blood cell count.
