# Supplementary figures and images for: Severe Painful Vaso-Occlusive Crises and Mortality in a Contemporary Adult Sickle Cell Anemia Cohort Study
Source: PLoS One. 2013 Nov 5;8(11):e79923. doi: 10.1371/journal.pone.0079923 (PMC3818240; doi:10.1371/journal.pone.0079923)

**
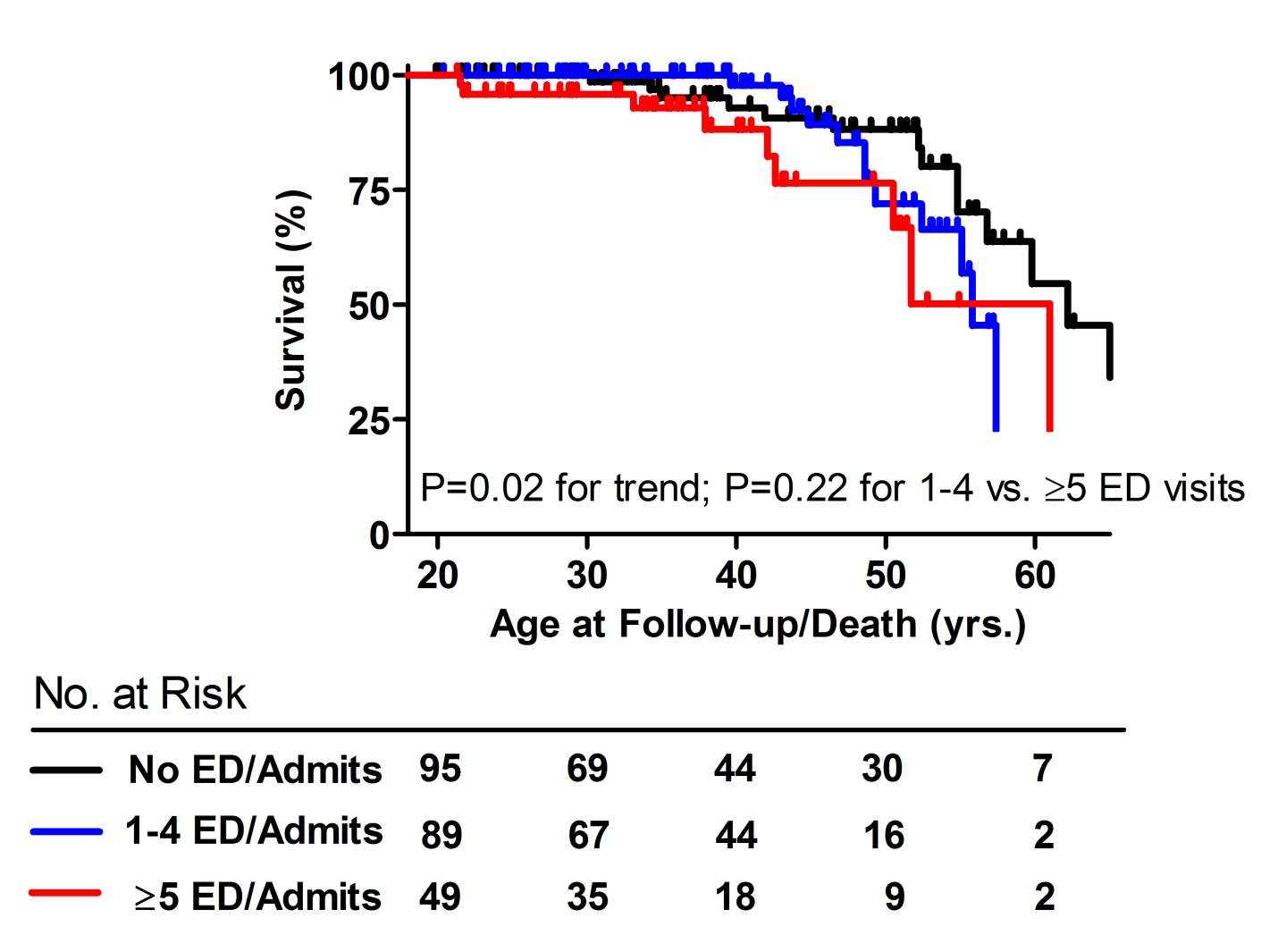
**

Supplement: Figure S1 — Kaplan Meier (KM) curve showing survival in sickle cell anemia is associated with the overall number of pain crisis events defined by ED visits and hospitalizations, but not sub-groups with more frequent occurrences. (DOCX) [file pone.0079923.s002.docx]
